# Use of Natural Language Processing of Patient-Initiated Electronic Health Record Messages to Identify Patients With COVID-19 Infection

**DOI:** 10.1001/jamanetworkopen.2023.22299

**Published:** 2023-07-07

**Authors:** Kellen Mermin-Bunnell, Yuanda Zhu, Andrew Hornback, Gregory Damhorst, Tiffany Walker, Chad Robichaux, Lejy Mathew, Nour Jaquemet, Kourtney Peters, Theodore M. Johnson, May Dongmei Wang, Blake Anderson

**Affiliations:** 1 Currently a medical student at Emory University School of Medicine, Atlanta, Georgia; 2 School of Electrical and Computer Engineering, Georgia Institute of Technology, Atlanta; 3 School of Computational Science and Engineering, Georgia Institute of Technology, Atlanta; 4 Division of Infectious Diseases, Emory University School of Medicine, Atlanta, Georgia; 5 Division of General Internal Medicine, Emory University School of Medicine, Atlanta, Georgia; 6 Department of Biomedical Informatics, Emory University School of Medicine, Atlanta, Georgia; 7 Emory University School of Medicine, Atlanta, Georgia; 8 Atlanta Veterans Affairs Healthcare System, Decatur, Georgia; 9 Department of Biomedical Engineering, Georgia Institute of Technology and Emory University, Atlanta, Georgia

## Abstract

**Importance:**

Natural language processing (NLP) has the potential to enable faster treatment access by reducing clinician response time and improving electronic health record (EHR) efficiency.

**Objective:**

To develop an NLP model that can accurately classify patient-initiated EHR messages and triage COVID-19 cases to reduce clinician response time and improve access to antiviral treatment.

**Design, Setting, and Participants:**

This retrospective cohort study assessed development of a novel NLP framework to classify patient-initiated EHR messages and subsequently evaluate the model’s accuracy. Included patients sent messages via the EHR patient portal from 5 Atlanta, Georgia, hospitals between March 30 and September 1, 2022. Assessment of the model’s accuracy consisted of manual review of message contents to confirm the classification label by a team of physicians, nurses, and medical students, followed by retrospective propensity score–matched clinical outcomes analysis.

**Exposure:**

Prescription of antiviral treatment for COVID-19.

**Main Outcomes and Measures:**

The 2 primary outcomes were (1) physician-validated evaluation of the NLP model’s message classification accuracy and (2) analysis of the model’s potential clinical effect via increased patient access to treatment. The model classified messages into COVID-19–other (pertaining to COVID-19 but not reporting a positive test), COVID-19-positive (reporting a positive at-home COVID-19 test result), and non–COVID-19 (not pertaining to COVID-19).

**Results:**

Among 10 172 patients whose messages were included in analyses, the mean (SD) age was 58 (17) years; 6509 patients (64.0%) were women and 3663 (36.0%) were men. In terms of race and ethnicity, 2544 patients (25.0%) were African American or Black, 20 (0.2%) were American Indian or Alaska Native, 1508 (14.8%) were Asian, 28 (0.3%) were Native Hawaiian or other Pacific Islander, 5980 (58.8%) were White, 91 (0.9%) were more than 1 race or ethnicity, and 1 (0.01%) chose not to answer. The NLP model had high accuracy and sensitivity, with a macro F1 score of 94% and sensitivity of 85% for COVID-19–other, 96% for COVID-19–positive, and 100% for non–COVID-19 messages. Among the 3048 patient-generated messages reporting positive SARS-CoV-2 test results, 2982 (97.8%) were not documented in structured EHR data. Mean (SD) message response time for COVID-19–positive patients who received treatment (364.10 [784.47] minutes) was faster than for those who did not (490.38 [1132.14] minutes; *P* = .03). Likelihood of antiviral prescription was inversely correlated with message response time (odds ratio, 0.99 [95% CI, 0.98-1.00]; *P* = .003).

**Conclusions and Relevance:**

In this cohort study of 2982 COVID-19–positive patients, a novel NLP model classified patient-initiated EHR messages reporting positive COVID-19 test results with high sensitivity. Furthermore, when responses to patient messages occurred faster, patients were more likely to receive antiviral medical prescription within the 5-day treatment window. Although additional analysis on the effect on clinical outcomes is needed, these findings represent a possible use case for integration of NLP algorithms into clinical care.

## Key Points

**Question:**

Can a natural language processing (NLP) model accurately classify patient-initiated electronic health record (EHR) messages and triage positive COVID-19 cases?

**Findings:**

In this cohort study of 10 172 patients, 3048 messages reported COVID-19–positive test results, and the mean (SD) message response time for patients who received treatment (364.10 [784.47] minutes) was faster than for those who did not (490.38 [1132.14] minutes). This novel NLP model classified patient messages with 94% accuracy and a sensitivity of 85% for messages that mentioned confirmed COVID-19 infection, discussed COVID-19 without mentioning a positive test result, or were unrelated to COVID-19.

**Meaning:**

These findings suggest that NLP-EHR integration can effectively triage patients reporting positive at-home COVID-19 test results via the EHR, reducing the time to first message response and increasing the likelihood of receiving an antiviral prescription within the 5-day treatment window.

## Teaser

This cohort study assesses the ability of a natural language processing model to classify patient-initiated electronic health record (EHR) messages and triage COVID-19 cases to reduce clinician response time and improve access to antiviral treatment.

## Introduction

The emergence and rapid spread of SARS-CoV-2 and subsequent variants has posed a unique threat to health care capacity worldwide. The COVID-19 pandemic has increased reliance on telemedicine and electronic health record (EHR) communications, as patient-initiated EHR message rates increased over 200%.^1^ Electronic messaging has the potential to improve clinician-patient communication, but high message volumes can impair efficiency and impose burdens on clinicians already experiencing burnout, ultimately resulting in patient morbidity from delayed responses.^2^

At-home rapid SARS-CoV-2 testing offers near-immediate results, increases testing access, and facilitates implementation of appropriate isolation measures without exposing clinicians and other patients.^3^ Many patients report positive SARS-CoV-2 test results to clinicians via patient-initiated EHR messages. When message responses are delayed due to increased EHR message burden, treatment windows may be missed.^4,5,6^ Oral antiviral therapies may decrease hospitalization, long-term sequelae, and death but have only been studied and authorized for use within 5 days of symptom onset.^3,7^ There are few examples of natural language processing (NLP) analysis of patient messages for use in clinical care.^8,9^

We developed an artificial intelligence solution to EHR message burden that rapidly identifies patient-authored messages reporting positive SARS-CoV-2 test results with the aim of facilitating timely administration of oral antiviral treatment. In a retrospective cohort analysis, we assessed whether there was an association between the time from when a patient sent a message reporting a positive test result to when their message was first opened by a member of their clinical team and whether the patient received antiviral treatment.

## Methods

In this cohort study, data from 5 Atlanta, Georgia, hospitals was approved by Emory University’s Institutional Review Board. The requirement for informed consent was waived based on negligible risk to patients and impracticality in obtaining consent from tens of thousands of patients.

We obtained internally completed SARS-CoV-2 polymerase chain reaction and rapid antigen test results and recorded external test results from Emory’s clinical data warehouse on a flow sheet via an honest broker informatician. A total of 187 217 messages sent by adult patients to their health care teams via a patient portal between March 30 and September 1, 2022, were analyzed. Race and ethnicity data were extracted from the EHR to characterize the study population and reduce uncontrolled confounding in model development and analysis. To train the model, a random sample of 14 537 messages were categorized by study clinicians as (1) mentioning confirmed COVID-19 infection (COVID-19 positive), (2) discussing COVID-19 without mention of a positive test result (COVID-19 other), or (3) unrelated to COVID-19 (non–COVID-19). We used transfer learning with a Bidirectional Encoding Representations of Transformers (BERT) NLP model^10^ to classify messages. The model, eCOV, was trained and validated on 14 537 clinician-labeled messages with a train to validation to testing ratio of 6:2:2; 5-fold cross-validation was applied to evaluate the performance across all samples in the testing set.^11^ All data were obtained and reported in concordance with the Strengthening the Reporting of Observational Studies in Epidemiology (STROBE) reporting guideline.

### Development and Evaluation of NLP Model

Several BERT models were deployed and tested, including the base BERT model,^10^ Bio ClinicalBERT,^12^ and distilBERT.^13^ The Bio ClinicalBERT model was initialized from the BioBERT model and pretrained on MIMIC-III (Medical Information Mart for Intensive Care III) clinical notes; the model shows performance superior to that of general-purpose embeddings on several clinical NLP tasks, such as the MedNLI (natural language inference) task annotated by physicians, nurses, and medical students and named entity recognition tasks. Meanwhile, distilBERT, a lightweight transformer model distilled from the base BERT model, preserved 95% performance in language understanding tasks with 40% fewer parameters and 60% faster computation. Using all 3 models yielded very similar classification performance. The language in messages from patients is similar to general-purpose text and very different from clinical notes; thus, the Bio ClinicalBERT model fails to show better classification performance than distilBERT for EHR text written by patients. Overall, the distilBERT model was found to be optimal for this specific application due to faster and more efficient computation.

To test the robustness of the distilBERT model, we conducted experiments to evaluate its classification performance with limited message samples. We divided the data set into a training and validation set of 10 000 samples and a holdout testing set of 4537 samples. We randomly extracted 1000 to 10 000 samples from the training and validation sets, with a 6:2 ratio of training to validation. We trained and evaluated each model on the holdout testing set. The plot shows that more training and validation samples led to better results, but even with just 1000 samples, the model achieved competitive performance. This finding is important because it suggests that clinicians can label a small number of messages and still achieve reasonable results when using the same NLP approach in a new hospital or for a new clinical task.

After clinical care for the episode in question had ceased, SQL (structured query language) queries were used to extract EHR data. Matching to chronic conditions, demographics, and medications, for example, was accomplished via matching of unique identifiers and was date matched to ensure medications and conditions were active during the clinical period of interest.

### Deployment of NLP Model

Once trained, the eCOV model’s 3-label classifier was prospectively deployed on 2907 new messages to evaluate accuracy. The first message reporting positive results for each patient was included; subsequent messages describing positive results within the study time frame were ignored.

A subset of messages representing patients self-reporting positive results was adjudicated by clinicians to determine whether the message was sent within the time frame of effectiveness for oral antivirals.^14^ The 2 primary outcomes were (1) physician-validated evaluation of the NLP model’s message classification accuracy and (2) analysis of the model’s potential clinical outcomes via increased patient access to treatment. Candidates without documented prescriptions for nirmatrelvir and ritonavir or for molnupiravir were identified as untreated, and those who had a prescription for nirmatrelvir and ritonavir or for molnupiravir within 5 days of the message creation date were identified as treated. Propensity score matching was performed to create similar classes to control for confounders. Logistic regression was performed on the COVID-19–positive group with the binary outcome of receiving or not receiving antiviral treatments in the 5-day window with covariates age, sex, White race, Charlson Comorbidity Index (CCI) score, and body mass index (BMI), similar to prior analyses of monoclonal antibody recipients.^15^ White race was a binary variable in this analysis because it was the most common race among the patient population.

### Evaluation of Model Accuracy

Clinician-assigned labels were compared with model-assigned labels to calculate the class-specific and weighted to unweighted mean of sensitivity (recall), specificity, precision, and F1 score. True-positive (TP), false-negative (FN), true-negative (TN), and false-positive (FP) labels were calculated for each class using a one-vs-rest approach. Class-specific sensitivity (recall) was calculated as TP/(TP + FN) and measured the proportion of patients with COVID-19 who were labeled as COVID-19 positive by the model. Class-specific specificity was calculated as TN/(TN + FP) and measured the proportion of patients without COVID-19 who were labeled as COVID-19 negative by the model. Class-specific precision was calculated as TP/(TP + FP) and measured the indications denoting, among all samples with positive test results, how many truly belonged to the target class. Class-specific F1 score was calculated as 2TP/(2TP + FP + FN). The F1 score is the arithmetic mean of precision and recall and is a balanced summarization metric of NLP model performance.^16,17^ The unweighted mean (macro mean) of each performance metric is the arithmetic mean of the above performance metrics across all classes, ignoring the number of samples of each class. The weighted mean of each performance metric includes the number of samples (support) in each class in calculation.

Time from message creation to first staff interaction, defined as the time when a clinical staff member first viewed the message, was calculated using clinical data warehouse timestamps. A paired *t* test was used to compare time to first message interaction between untreated and treated patients. The Figure depicts a flowchart of study methods, including NLP model training and evaluation as well as cohort study analyses. Two-sided α = .05 indicated statistical significance.

**Figure.**
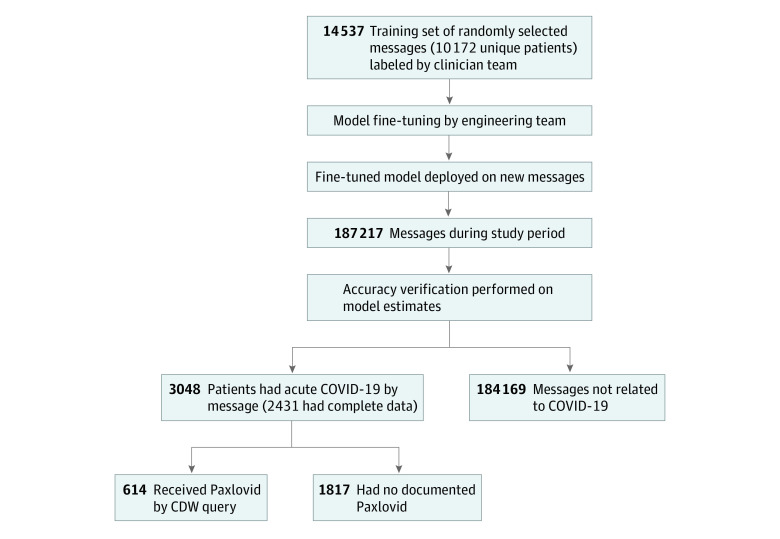
Study Flowchart CDW indicates clinical data warehouse.

## Results

Of the 10 172 patients whose messages were included in analyses, the mean (SD) age was 58 (17) years; 6509 (64.0%) were women and 3663 (36.0%) were men. In terms of race and ethnicity, 2544 patients (25.0%) were African American or Black, 20 (0.2%) were American Indian or Alaska Native, 1508 (14.8%) were Asian, 28 (0.3%) were Native Hawaiian or other Pacific Islander, 5980 (58.8%) were White, 91 (0.9%) were more than 1 race or ethnicity, and 1 (0.01%) chose not to answer. Table 1 gives complete demographic information. Overall, 3048 of 187 217 incoming patient-generated messages (1.6%) were related to self-reported positive SARS-CoV-2 test results and, of those, 2982 (97.8%) were not otherwise documented in structured EHR data. An evaluation of 50 patients’ external pharmacy records in the EHR showed concordance between internal and external antiviral prescriptions in 45 (90.0%) of the 50 patients examined. The 3048 patients who sent messages reporting COVID-19 diagnoses that were not recorded in structured EHR data were evaluated in retrospective cohort analysis. The eCOV model’s macro F1 score was 94%, with individual class F1 scores of 87% for COVID-19–other, 95% for COVID-19–positive, and 100% for non–COVID-19 messages. Sensitivities were 85% for COVID-19–other, 96% for COVID-19–positive, and 100% for non–COVID-19 messages (Table 2).

**Table 1.** Demographic Characteristics for Patient Sample Whose EHR Messages Were Used to Train the Model

Characteristic	No. (%) of patients (N = 10 172)
Age, y	
18-30	615 (6.0)
31-40	1327 (13.0)
41-50	1544 (15.2)
51-60	1865 (18.3)
61-70	2162 (21.3)
71-80	1946 (19.1)
81-90	713 (7.0)
Sex	
Men	3663 (36.0)
Women	6509 (64.0)
Race or ethnicity	
African American or Black	2544 (25.0)
American Indian or Alaska Native	20 (0.2)
Asian	1508 (14.8)
Native Hawaiian or other Pacific Islander	28 (0.3)
White	5980 (58.8)
Multiple races or ethnicities	91 (0.9)
Chose not to answer	1 (0.01)

Abbreviation: EHR, electronic health record.

**Table 2.** Sensitivity (Recall), Specificity, Precision, and F1 Score of the eCOV Model^a^

Message category	No. (%) of patients	Sensitivity, %	Specificity, %	Precision, %	F1 score, %
COVID-19 other	148 (5.1)	85	100	90	87
COVID-19 positive	307 (10.6)	96	99	93	95
Non–COVID-19	2452 (84.4)	100	99	100	100
Unweighted mean	NA	94	99	94	94
Weighted mean	NA	99	99	99	99

Abbreviation: NA, not applicable.

^a^ Class-specific sensitivity was calculated as true positive divided by true positive plus false negative [(TP)/(TP + FN)] in a one-vs-rest approach; class-specific specificity, true negative divided by true negative plus false positive [TN/(TN + FP)] in a one-vs-rest approach; class-specific precision, TP/(TP + FP) in a one-vs-rest approach; and class-specific F1 score, 2TP/(2TP + FP + FN) in a one-vs-rest approach. The unweighted mean (macro mean) of each value was the arithmetic mean. The weighted mean of each value included the number of patients for each class into calculation.

The treated and untreated groups had significant differences in age, BMI, and CCI (Table 3). Mean (SD) response time was significantly faster for the treated group (364.10 [784.47] minutes) than the untreated group (490.38 [1132.14] minutes; *P* = .03). Logistic regression showed an association between shorter response time and likelihood of receiving an antiviral prescription (odds ratio, 0.99 [95% CI, 0.98-1.00]; *P* = .003) when controlling for age, BMI, CCI, male sex, and White race (Table 4).

**Table 3.** Differences Between COVID-19–Positive Patients by Antiviral Treatment^a^

Characteristic	Patient group, mean (SD)		*P* value^b^
	Did not receive antiviral treatment	Received antiviral treatment	
Age, y	54.48 (15.90)	56.10 (15.37)	.01
BMI	28.79 (6.67)	29.59 (6.77)	.02
CCI score	2.71 (3.06)	3.05 (2.94)	.01
Minutes to first interaction	490.38 (1132.14)	364.10 (784.47)	.03

Abbreviations: BMI, body mass index (calculated as the weight in kilograms divided by the height in meters squared); CCI, Charlson Comorbidity Index.

^a^ Algorithm was created from 3 years of outpatient diagnoses and problem list diagnosis matching. The BMI was the most recent measured before the message was sent. Minutes to first interaction was defined as time from the message created by the patient to first staff interaction.

^b^ Calculated using a paired *t* test; the significance threshold was α = .05.

**Table 4.** Differences Between COVID-19–Positive Patients Who Received Antiviral Treatment and Those Who Did Not^a^

Characteristic	Odds ratio (95% CI)	*P* value^b^
Age	1.01 (1.00-1.01)	.001
BMI	1.02 (1.01-1.03)	.001
CCI	1.02 (1.00-1.07)	.03
Minutes to first interaction	0.99 (0.98-1.00)	.003
Male sex	1.20 (0.95-1.38)	.15
White race	0.87 (0.68-1.12)	.45

Abbreviations: BMI, body mass index; CCI, Charlson Comorbidity Index.

^a^ Algorithm was created from 3 years of outpatient diagnoses and problem list diagnosis matching. The BMI was the most recent measured before the message was sent; minutes to first interaction, time from the message created by the patient to first staff interaction.

^b^ Calculated using logistic regression; the significance threshold was α = .05.

## Discussion

Electronic health record messaging offers convenient patient self-reporting of COVID-19 test results to clinicians, but high message volumes, demands on clinician time, and lack of documentation into structured EHR data elements are barriers to timely treatment. The eCOV model is the first clinically deployable NLP model to triage incoming, self-reported positive COVID-19 cases in real time. The eCOV model identified acute COVID-19 cases from patient-initiated EHR messages with 94% sensitivity.

For large health care systems receiving thousands of messages daily, the only viable option for triaging clinically urgent messages is a technological one. Whether a physician acts on a message the day it is sent or multiple days later might determine whether oral antiviral treatment can be appropriately administered and whether benefits of reduced risk of hospitalization or mortality can be realized.^18^ This remains critically important as concurrent COVID-19, influenza, and respiratory syncytial virus epidemics threaten hospital capacity. This method also allows for more accurate reporting of positivity, as only 66 cases identified in our cohort (2.2%) were otherwise documented in the structured data elements more easily accessed via traditional EHR database queries.

The availability and convenience of at-home SARS-CoV-2 tests makes them an attractive option for patients. Patients with positive test results for COVID-19 at home and who experience severe symptoms can then send an EHR message to their primary care physician reporting the positive test results and inquiring about treatment options. Some patients can then be adequately treated remotely by their primary care physician, reducing hospitalization rates, infection risk for health care workers, and burden on the medical care system. However, this requires initiation of treatment in a timely fashion, and the large burden of EHR tasks, including patient-initiated messages, makes it difficult for physicians to rapidly respond to all patient messages, many of which are not urgent. Patients whose messages have slower response times are less likely to receive an antiviral prescription within the 5-day treatment initiation window. The eCOV model accurately and instantaneously identifies and triages patient-initiated messages reporting positive COVID-19 test results. By classifying patient messages accurately and improving the speed of treatment access, NLP, when integrated into the EHR, has the potential to improve clinical outcomes while simultaneously reducing health care system burden. Additional analyses on outcomes following clinical integration are needed to quantify true clinical impact.

### Limitations

We found an association between longer response time and absence of antiviral prescription. Although propensity score matching was performed to create similar classes, factors such as age and preexisting medical conditions may contribute to the decision to prescribe antivirals independent of response time. Additionally, some patients may have received treatments other than oral antivirals that were not captured, such as monoclonal antibody therapies or intravenous remdesivir, during the treatment window. There are a multitude of factors that contribute to treatment decisions—including patient preference, vaccination status, insurance status, and cost of medications—that were not captured in this analysis. Further research is required to determine a causal relationship between response time and treatment. Additional limitations of this study include absence of visual validation of test results and inability to systematically verify prescriptions from other facilities or treatment adherence. However, underreporting rates likely significantly outweigh false-positive reporting.^3,19^ Misclassification based on external prescription of antivirals not identified by our database is possible but likely small based on evaluation of patients’ external pharmacy records in the EHR, which showed concordance between internal and external prescriptions in 45 (90.0%) of 50 patients examined. Additionally, symptom onset could not be systematically evaluated, so a subset of patients identified as antiviral candidates may have been out of the treatment window by the time a message was sent.

## Conclusions

The findings of this cohort study suggest that nearly 98% of patients reporting their positive at-home COVID-19 test results were not otherwise documented as SARS-CoV-2 positive in the EHR. These represent opportunities for patients whose messages are at risk of being missed among high volumes of EHR communications to receive oral antivirals within the 5-day treatment window. The eCOV model uses NLP to classify messages with high accuracy and can be deployed as an automated triage tool to facilitate timely identification of treatment candidates. Although additional analysis of the effect on clinical outcomes is needed, these findings represent a possible use case for integration of NLP algorithms into clinical care.

## Notes

Supplement.
Data Sharing Statement
