# Supplementary material for: Use of Natural Language Processing of Patient-Initiated Electronic Health Record Messages to Identify Patients With COVID-19 Infection
Source: JAMA Netw Open. 2023 Jul 7;6(7):e2322299. doi: 10.1001/jamanetworkopen.2023.22299 (PMC10329205; doi:10.1001/jamanetworkopen.2023.22299)
Supplement: Supplement. — Data Sharing Statement [file jamanetwopen-e2322299-s001.pdf]

Data Sharing Statement

Mermin-Bunnell. Use of Natural Language Processing of Patient-Initiated Electronic Health Record Messages to Identify Patients With COVID-19 Infection. *JAMA Netw Open*. Published July 07, 2023. doi:10.1001/jamanetworkopen.2023.22299

Data
Data available: No

Additional Information
Explanation for why data not available: It is extremely difficult to fully deidentify free text and as such it is not allowed by our institution.
